# FISH+ is a ready-to-use proximity labeling method for simultaneous RNA visualization and RNA-interacting protein identification

**DOI:** 10.1038/s42003-026-10509-0

**Published:** 2026-06-23

**Authors:** Mingxing Lu, Fenglin Shen, Yun Liu, Xianghuo He

**Affiliations:** 1 Fudan University Shanghai Cancer Center and Institutes of Biomedical Sciences, Department of Oncology, Shanghai Medical College, Fudan University, Shanghai, China; 2 Institutes of Biomedical Sciences, Fudan University, Shanghai, China; 3 MOE Key Laboratory of Metabolism and Molecular Medicine, Department of Biochemistry and Molecular Biology, School of Basic Medical Sciences and Shanghai Xuhui Central Hospital, Fudan University, Shanghai, China

**Keywords:** Molecular imaging, RNA probes, Synthetic biology

## Abstract

Traditional proximity biotinylation approaches require extensive genetic engineering or intricate purification steps. Here, we introduce FISH+, a ready-to-use RNA proximity labeling method that relies on the recruitment of peroxidase to RNA targets, and facilitates in situ proximity biotinylation in fixed cells. This method permits concurrent visualization of RNA molecules and identification of RNA-interacting proteins. Using this method, we visualized 45S and NEAT1 RNA, and captured their proximal proteins, demonstrating the capability to concurrently visualize RNA and identify proximal proteins. By targeting PNCTR (~36 copies per cell), we observed distinctly bright RNA dots, representing the combined biotinylation signals from both RNA and proximal proteins in situ. This indicates the potential of the FISH+ method for enhanced RNA visualization. We further generalized the FISH+ method to explore XIST-interacting proteins, a number of reported interactors were significantly enriched, such as SPEN, CIZ1 and RBM15. Using quantitative mass spectrometry, we show that FISH+ correctly identifies known RNA-protein interactions in the nucleus of human cells. Overall, we established a watch-and-catch punctate RNA method through the integration of RNA fluorescence in situ hybridization (FISH) with proximity biotinylation. This method provides additional spatial information for the characterization of RNA-centric interactions in fixed, genetically unperturbed samples.

## Abstract (web summary)

FISH+ extends RNA FISH from visualization to protein identification through a simple, robust and highly accessible “watch-and-catch” proximity labeling method for punctate RNAs.

## Introduction

RNA biology is coordinated by the dynamic interactions of RNAs and RNA-binding proteins (RBPs)^1,2^. These RNA-protein interactions are pervasive in cells. However, RNA-centric methods for interaction detection frequently meet various obstacles or inefficiency^3,4^. Traditional RNA-centric methods to capture RNA-protein interactions are mainly based on complementary synthetic oligonucleotides, which target a given RNA and capture its binding proteins in vitro^5,6^. The advent of programmable RNA-targeting CRISPR-Cas13 systems allows researchers to manipulate specific RNAs in live cells^7,8^. However, these methods suffer from limited detection sensitivity.

RNA in situ hybridization has been widely used to visualize specific RNAs in cells. We combined RNA in situ hybridization with proximity labeling to detect proteins associated with specific RNAs in their native cellular environment. Proximity labeling is a powerful technology to identify proximal proteins to a bait of interest despite obstacles such as protein insolubility and transient or weak interactions^9^, which are refractory to conventional biochemical approaches. Currently, several proximity biotinylation enzymes, including bioID, TurboID, APEX2, and HRP, have been engineered for diverse biological settings^10^. In this study, we employed HRP as it offers rapid proximity biotinylation.

However, current proximity labeling methods typically involve fusing the proximity biotinylation enzyme to a bait of interest through either CRISPR-based knock-in approach or plasmid-based expression in live cells. Genetic manipulation of target cells to incorporate the proximity biotinylation enzyme is labor-intensive, and the fusion of the enzyme to a bait of interest may perturb its native function. To address these limitations in protein analysis, Bar et al. recently demonstrated a strategy for antibody-mediated proximity labeling in fixed cells, enabling protein-centric mapping without genetic engineering^11^. Here, we present FISH+, an RNA-centric reciprocal to this approach.

To simplify proximity labeling workflows in fixed cells, we introduce a commercially available proximity labeling fusion protein called streptavidin-HRP. This fusion protein consists of streptavidin conjugated to the HRP proximity biotinylation enzyme. Leveraging the superb binding affinity between streptavidin and biotin (${K}_{d}\approx {10}^{-15}{{\rm{M}}}$), along with biotin-labeled probe hybridization, the streptavidin-HRP can be directed to RNAs of interest and biotinylate the RNA local environments within cells. Through biotinylation labeling, we are able to concurrently visualize RNA and identify RNA-proximal proteins in situ. This method represents a ready-to-use proximity biotinylation procedure that provides additional spatial information for the characterization of RNA-centric interactions in fixed, genetically unperturbed samples.

## Results

### The development of FISH+

**Fig. 1:**
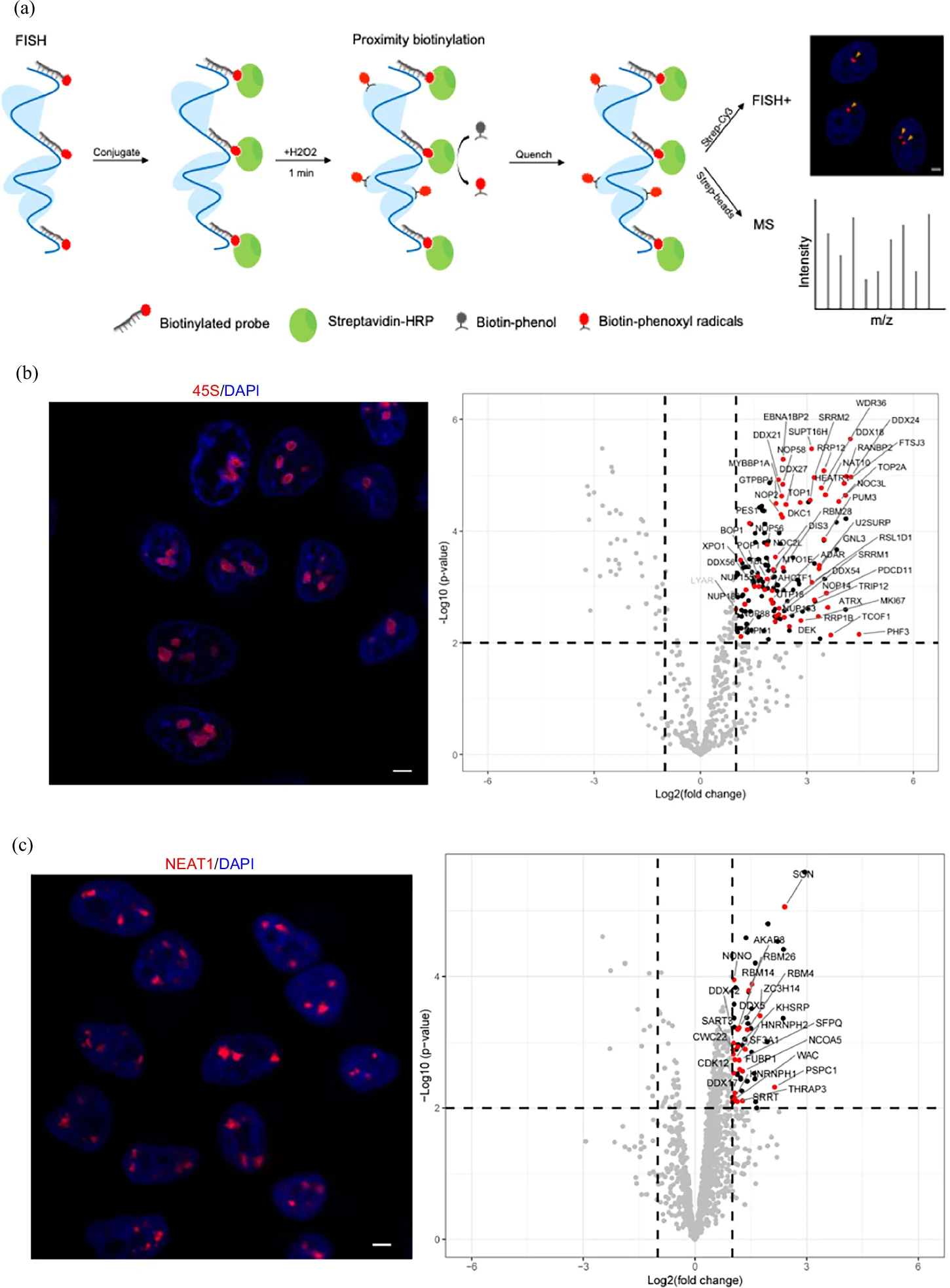
Ready-to-use RNA proximity biotinylation in fixed cells. **a** Schematic illustration of the FISH+ working principle. Biotinylated oligonucleotide probes recruit streptavidin-HRP to the RNA of interest to direct proximity biotinylation. The representative image shows strep-Cy3 staining (red), indicative of FISH+ labelling, with specific signals highlighted by orange arrowheads. The schematic also illustrates the alternative workflow, where biotinylated proteins are affinity-enriched using strep-beads and analyzed through mass spectrometry (MS). Strep-Cy3: streptavidin-Cy3, strep-beads: streptavidin-beads. **b**, **c** Biotinylation of proteomes associated with 45S and NEAT1 RNAs. (left) Representative images showing RNA visualization, Scale bar: 10 µm. (right) Volcano plots of proteins identified by FISH+ in Hela cells. For both 45S and NEAT1, significantly enriched proteins (*p*-value < 0.01, fold change > 2) are shown as black dots (*n* = 3 for each RNA and non-targeting control). Red dots represent known RBPs for 45S and NEAT1, compiled from reported literature and the StarBase v2.0 database. FC fold change between target and non-targeting control.

Here we present a ready-to-use proximity biotinylation protocol utilizing RNA in situ hybridization. The approach commences with the hybridization of biotin-labeled probes to the target RNAs. Subsequently, streptavidin-HRP is recruited to these RNAs. In the presence of biotin-phenol and H_2_O_2_, streptavidin-HRP catalyzes the in situ biotinylation of proteins proximate to the targeted RNAs. The biotinylated proteins are then detectable with streptavidin-Cy3, along with the target RNAs (Fig. 1a, FISH+). Additionally, the biotinylated proteins can be isolated using streptavidin beads and subsequently analyzed through mass spectrometry (Fig. 1a, MS). With the high sensitivity of HRP as a proximity labeling enzyme (Supplementary Fig 1), we established a ready-to-use RNA proximity biotinylation method, termed FISH+, through the integration of RNA fluorescence in situ hybridization (FISH) with proximity biotinylation. This method enables the concurrent visualization of RNA and the identification of RNA-associated proteins within fixed cells.

### Evaluation of FISH+ with known RNA-protein interactions

We first evaluate the FISH+ method by targeting the 45S ribosomal precursor RNA in Hela cells. Biotin-labeled probes guided streptavidin-HRP to the 45S RNA to catalyze proximity labeling. Detection via streptavidin-Cy3 revealed that the biotinylation signal localized to distinct foci within the cell nucleus (Fig. 1b). We further validated the method by targeting NEAT1 RNA, also in Hela cells. Similar to the 45S experiments, NEAT1 targeting resulted in clearly defined nuclear puncta (Fig. 1c).

We next characterized the protein interactomes of 45S and NEAT1 RNAs. Biotinylated proteins were enriched by streptavidin beads, and the proteomes in proximity to the targeted RNAs were analyzed by label-free quantitative mass spectrometry in triplicate. Analysis of the proteins adjacent to 45S RNA identified established nucleolar markers, including DKC1, NOP2, NOP58, NOP56, TCOF1, and PDCD11. Similarly, the NEAT1-associated proteome included well-known NEAT1-interacting proteins such as NONO, KHSRP, RBM14, and SFPQ. We also identified numerous paraspeckle markers, including AKAP8, ZC3H14, PSPC1, consistent with the role of NEAT1 as a structural scaffold for paraspeckles^12^. We then performed gene ontology enrichment analysis of 45S and NEAT1 interactomes. We observed enrichment of nucleolus-specific terms for 45S and RNA processing-related terms for NEAT1 (Suppl. Fig 2). This functional profiling aligns with the known regulatory roles of the target RNAs, confirming the specificity of the identified candidates. Collectively, these experimental results demonstrate the efficacy of FISH+ as a robust method for concurrent in situ visualization of RNA and identification of RNA-binding proteins.

### Optimization of proximity labeling signals

**Fig. 2:**
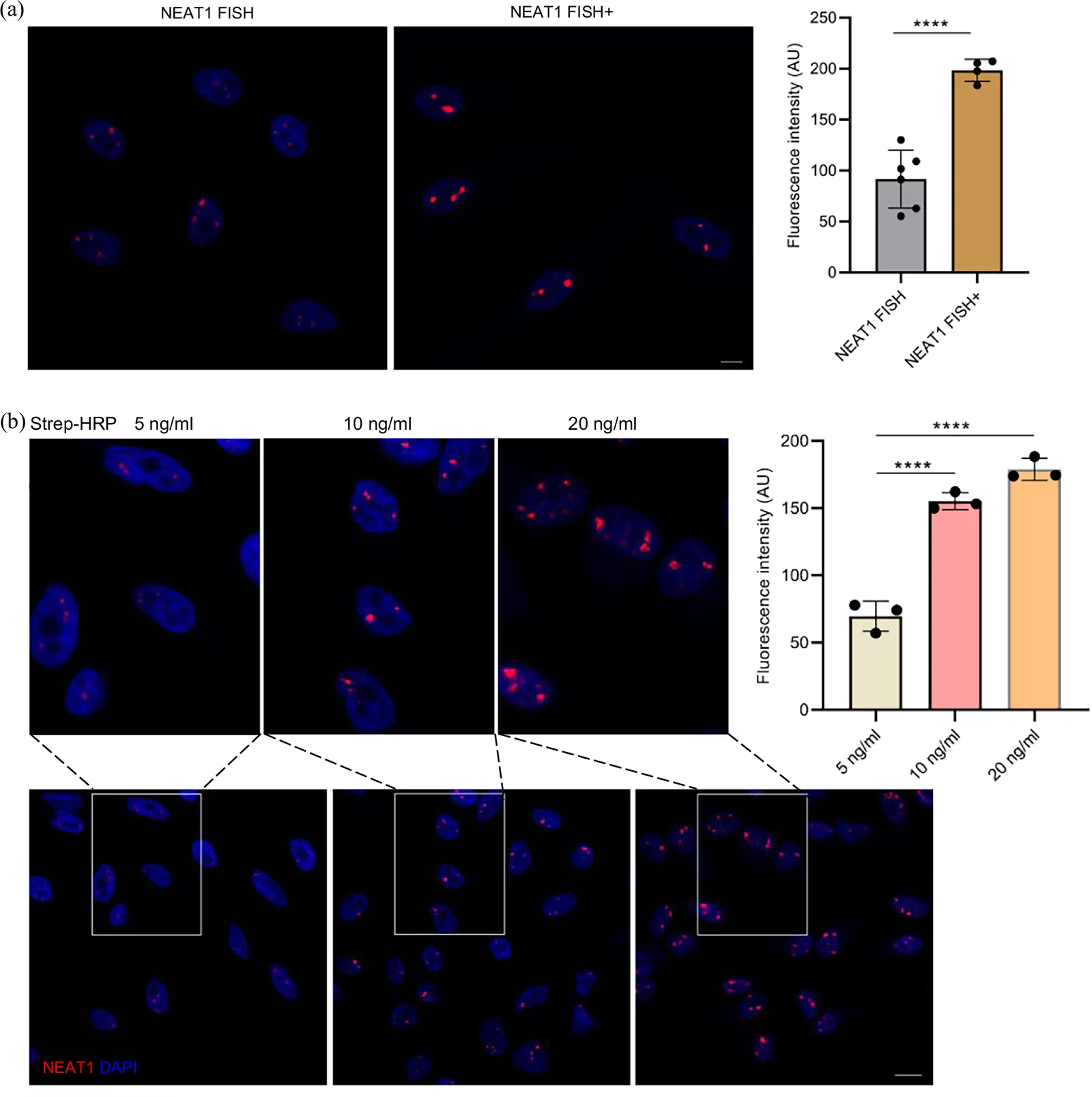
Signal intensity of RNA labeling is affected by labeling enzyme concentration. **a** FISH+ signal is stronger than FISH signal. Scale bars: 25 µm. Arbitrary units, AU. Images are represented as the mean ± SD (FISH *n* = 6; FISH+ *n* = 4). Unpaired t-test. **b** Optimization of streptavidin-HRP concentration. The signal intensity of RNA labeling increases with higher enzyme concentrations. However, excess HRP leads to an expanded signal radius and reduced spatial precision. These results indicate that 10 ng/ml streptavidin-HRP for NEAT1 RNA provides the optimal balance between signal intensity and spatial resolution. streptavidin-HRP: strep-HRP. Scale bars: 50 µm. Images are represented as the mean ± SD (*n* = 3). One-way ANOVA with multiple comparisons correction. ns not significant, *p < 0.05, **p < 0.01, ***p < 0.001, ****p < 0.0001.

We first compared FISH+ to standard FISH using NEAT1 RNA as a model system. Consistent with the proximity labelling mechanism, FISH+ generated significantly stronger signals than standard FISH (Fig. 2a). This signal enhancement is attributed to the fact that the final visualization represents a composite signal derived from both the RNA-hybridized probes and the biotinylated proximal proteins. We validated this protein-specific labelling through nuclease digestion assays: while RNase A treatment effectively degraded RNA, the FISH+ biotinylation signal persisted (Suppl. Figs. 3, 4). This confirms that FISH+ captures a stable protein “footprint” of the RNA granule, which remains detectable even after the underlying RNA scaffold is removed.

To ensure high labeling specificity, we further optimized the probe design and adjusted the labeling enzyme concentration. We utilized oligonucleotide probes designed via the Stellaris Probe Designer, which have been validated in prior studies^12–14^. Subsequently, we evaluated a concentration series of streptavidin-HRP to determine the optimal balance between detection sensitivity and spatial precision (Fig. 2b). We observed that the signal radius expanded with increasing enzyme concentration—a characteristic of HRP-mediated radical deposition. Specifically, an excess of HRP (as seen at 20 ng/ml) generates a surplus of radicals that expands the deposition radius, artificially enlarging the fluorescent puncta and potentially causing false-positive proximal interactions. Conversely, 5 ng/ml yielded insufficient detection sensitivity. We therefore selected 10 ng/ml as the optimal concentration for NEAT1, as it yielded distinct, resolvable granules without over-saturation. We note that the optimal working concentration may depend on endogenous target RNA abundance and should be determined empirically for different targets.

### PNCTR RNA visualization with FISH+

**Fig. 3:**
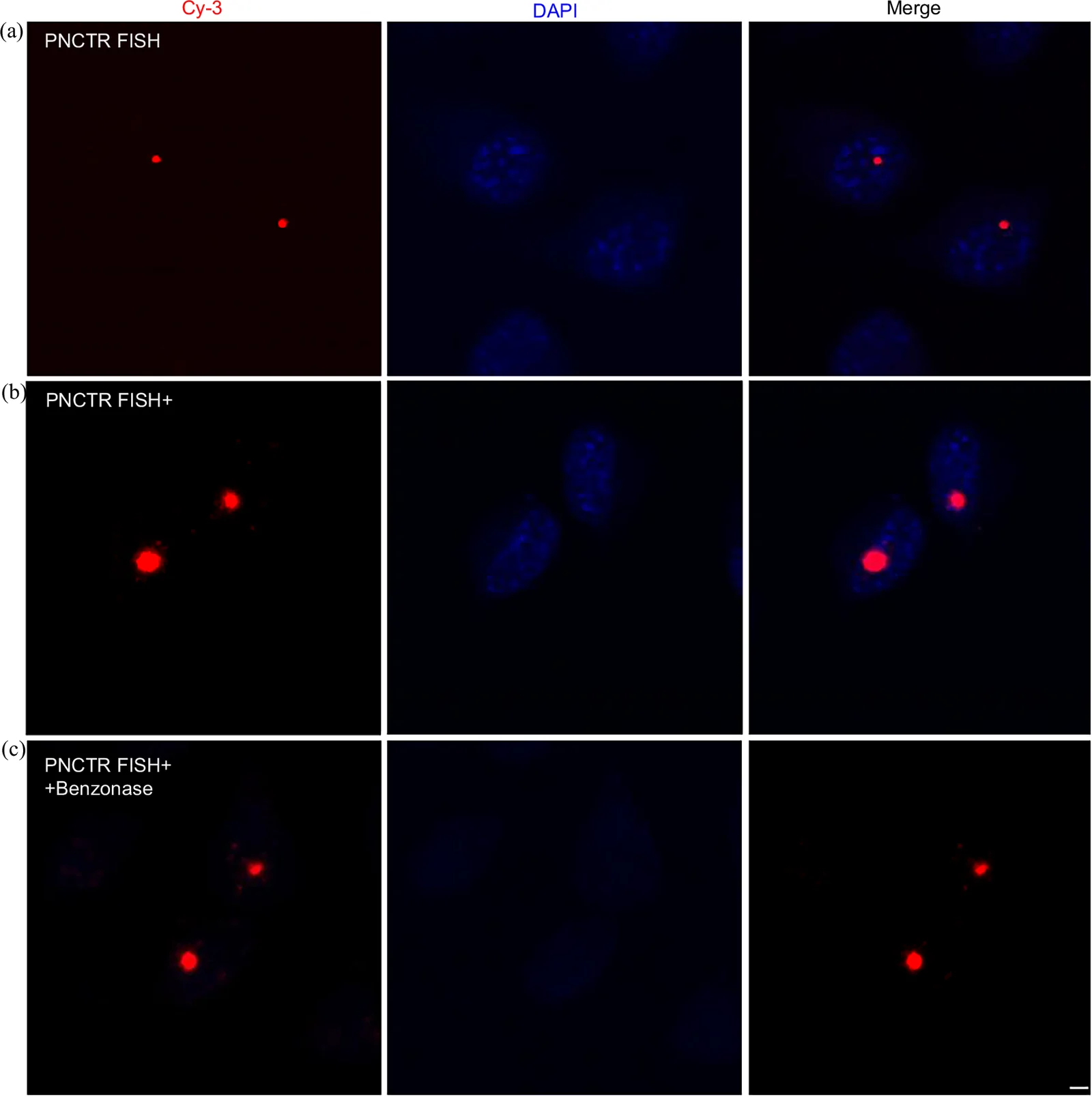
PNCTR RNA FISH+. **a** RNA FISH detection of PNCTR in Hela cells shows sparse puncta signals within the nucleus. **b** RNA FISH+ detection of PNCTR produces brighter and more prominent puncta, consistent with amplification of the RNA-associated signals through proximity labeling. **c** Following Benzonase treatment, nuclear nucleic acids were removed, as evidenced by loss of DAPI staining, whereas the PNCTR-associated FISH+ signal remained detectable. This result indicates that FISH+ labels proteins in proximity to PNCTR RNA and that the retained signal reflects PNCTR-proximal protein labeling rather than intact nucleic acid staining. Scale bars: 10 µm.

We next employed the Pyrimidine-rich Non-Coding Transcript (PNCTR) to evaluate the detection sensitivity of FISH+. PNCTR functions by sequestering the splicing factor PTBP1 within the perinucleolar compartment (PNC), thereby modulating PTBP1-mediated pre-mRNA splicing. Previous studies estimate that a typical Hela cell expresses ~36 copies of PNCTR^15^, making it an idea candidate for testing sensitivity. While standard RNA FISH revealed only sparse and faint dots within Hela nuclei, FISH+ significantly intensified the PNCTR signal (Fig. 3). To further confirm that this amplification was protein-dependent, we treated samples with Benzonase. Consistent with our observations for 45S and NEAT1 (Suppl. Fig. 5), the PNCTR signals persisted following Benzonase digestion, despite the complete absence of DAPI signals. We subsequently performed PNCTR FISH+ mass spectrometry, which captured the well-known interactor PTBP1 (Suppl. Fig. 6). These results demonstrate the capability of the FISH+ method to visualize proximal proteins around RNA targets, confirming that the enhanced signal is a composite derived from both the RNA-hybridized probes and the surrounding biotinylated proteins.

### Profiling of RNA-binding proteins via FISH+

**Fig. 4:**
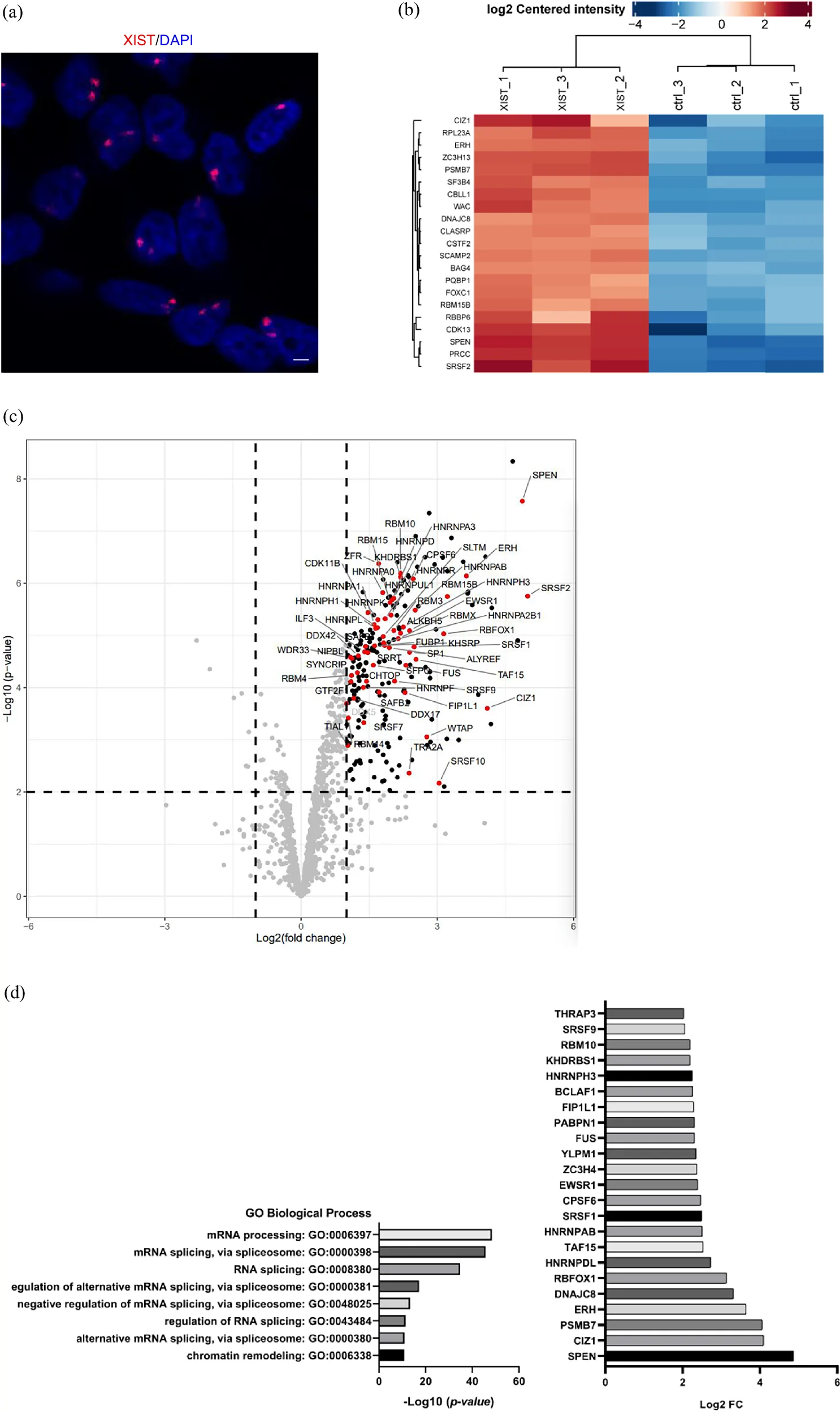
XIST-associated proteins in 293 T cell. **a** Biotinylation of proteomes associated with XIST dots. RNA visualization, Scale bar: 10 µm. **b** Log2-centered intensity heatmap displaying the top differentially enriched proteins. Affinity enrichments were performed in biological triplicates (XIST: XIST_1, XIST_2, XIST_3; non-targeting controls: ctrl_1, ctrl_2, ctrl_3). The full heatmap is provided in Suppl. data 3. **c** Volcano plot analysis of XIST-associated proteins identified by FISH+ in Hela cells. The plot compares XIST capture versus non-targeting controls (*n* = 3). Significantly enriched proteins (*p*-value < 0.01, fold change > 2) are indicated by black dots. Known XIST-interacting RBPs, compiled from reported literature and StarBase v2.0 database, are highlighted in red. FC, fold change of target and non-targeting control. **d** Top 8 significant Gene Ontology terms for XIST-associated proteins on the DAVID website. Top 23 significantly enriched proteins are listed.

Having established the ready-to-use RNA proximity biotinylation strategy via the streptavidin-HRP enzyme in fixed cells, we next generalized the FISH+ method to explore XIST-interacting proteins. XIST plays a key role in X chromosome inactivation during early embryonic development and dysregulation of XIST has been linked to various diseases^16^. In HEK293T cells, XIST expression appears as 1~2 dots per cell in nuclei (Fig. 4a). The proteomic experiment using the FISH+ method identified 2785 proteins with at least 2 unique peptides. For enrichment analysis, we applied label-free mass spectrometry quantification with a cut-off of more than twofold enrichment and an adjusted *p*-value < 0.01 (Fig 4b, c). 219 candidates were significantly enriched shown as black dots in the XIST FISH+. Among these, 59 proteins were previously reported XIST-interacting proteins from the literature^5,6,17^ and the StarBase v2.0 database, indicated by the red dots.

Furthermore, we found that 23 XIST-interacting proteins were significantly enriched with a more stringent cut-off (*p*-value < 0.01, fold change > 2). Many of these are well-known XIST-interacting proteins, such as the signature XIST-binding protein SPEN, as well as CIZ1 and RBM15, demonstrating the robust reproducibility of the FISH+ method. Gene ontology (GO) analysis for the significantly enriched candidates showed that XIST-interacting proteins were largely involved in RNA splicing and chromatin remodelling (Fig. 4d).

## Discussion

In this work, we developed FISH+, a ready-to-use RNA proximity biotinylation method that leverages streptavidin-biotin conjugation to recruit peroxidase to RNAs of interest in fixed, genetically unperturbed cells. Unlike most proximity-labeling methods, which rely on the expression of recombinant enzymes in live cells, FISH+ utilizes commercially available streptavidin-HRP to target RNA molecules via biotin-labeled probes. This design also eliminates the need for complex protein engineering, such as APEX2 fusion^18^ or extensive purification required by previous hybridization-based method^14^. Consequently, FISH+ provides a streamlined workflow that potentially expands the applicability of in situ RNA proximity biotinylation to diverse specimens.

The core mechanism of FISH+ exploits the high binding affinity of streptavidin and biotin, allowing the assay to tolerate high-stringency washing conditions while maintaining a low background signal. By integrating RNA in situ hybridization with proximal biotinylation, the method concurrently labels the target RNA and biotinylates its proximal protein environment. This dual labelling achieves synergistic signal amplification that effectively “hits two birds with one stone”, enabling the visualization of RNA-protein interactions with high sensitivity. Analogous to optimizing exposure in TSA-based Western blots, the sensitivity of FISH+ can be fine-tuned by adjusting enzyme concentration and incubation time to suit the abundance of the target RNA.

We validated the FISH+ design by targeting 45S, NEAT1, PNCTR, and XIST RNAs. Mass spectrometry analysis confirmed that the most significant RNA-interacting proteins identified had been previously characterized in the literature^14,19,20^. To rigorously assess sensitivity, we cross-referenced our data with known interacting RBPs (Suppl. Fig. 7). While FISH+ captured the majority of canonical binders, some known RBPs did not meet our strict enrichment criteria. For instance, the protein FBL, a known partner of 45S rRNA, was detected in our raw mass spectrometry data but did not pass the significance cut-off to be labeled as “enriched” relative to the control. We reason that such discrepancies may be attributed to differences in cellular context or the dynamic nature of specific RNA-protein interactions.

Despite its advantages, the current iteration of FISH+ presents certain limitations. First, the efficacy of the method depends heavily on probe specificity and target RNA abundance. While the enzymatic amplification facilitates detection, targeting low-abundance RNAs remains challenging and may result in reduced signal-to-noise ratios if non-specific streptavidin-HRP binding occurs. Second, the relatively large size of the streptavidin-HRP conjugate (~100 kDa) represents a potential constraint, as it may impose steric hindrance and restrict access to densely compacted molecular complexes. Third, FISH+ cannot differentiate between direct RNA-binding proteins and those that associate indirectly via protein complexes. Further validation may be necessary. Nevertheless, FISH+ offers a versatile, ready-to-use strategy for concurrent visualization of RNA molecules and identification of their interactors simply by using FISH probe to target the RNA of interest.

## Methods

### Cell culture

Hela cells and HEK-293T cells were obtained from China National Collection of Authenticated Cell Culture. Cells were maintained in DMEM (Gibco) supplemented with 10% fetal bovine serum (Biological Industries, BI-04-001-1C), penicillin, and streptomycin at 37 °C with 5% CO_2_ in cell culture incubator.

### Reagents and antibodies

Dynabeads MyOne Streptavidin C1(life technologies 65002). Ribonucleoside vanadyl complexes (Sigma, R3380-5ML). biotintyramide (Iris BiotechGmbH, LS-3500). Benzonase (Beyotime biotechnology, D7121-25KU), Murine RNase Inhibitor (Vazyme, R301-03) Nuclease-Free Water (Invitrogen, AM9930), Ambion Formamide (Deionized), Catalog number: AM9342. Triton X-100 (Sigma, T8787-50ML) Iodoacetamide (Beyotime ST1411-5g), Dithiothreitol (Beyotime, ST040-5g). Pierce™ 16% Formaldehyde (w/v), Methanol-free (Thermo Scientific, 28906).

### In situ hybridization oligonucleotide probes

The used probes in this study were designed using the Stellaris Probe Designer online tool, checked through BLAST searches against NCBI, and tested via RNA FISH. Additionally, we consulted previously published literature to select appropriate probe sets (Suppl. data 1). To ensure the specificity of the FISH probe sets, the synthesized probes were divided into two subset pools: “even” and “odd” pool of probes. For a probe set to be considered valid, the signals from these two subsets should largely display a similar localization pattern to the full probe set^21,22^. For instance, we divided the MALAT1 FISH probe set into odd- and even-number pools. The resulting images showed that the signal pattern from both subsets closely matched the full probe set (Suppl. Fig. 8), confirming the specificity of the designed probes. We used scrambled sequences or the same biotinylation steps, omitting the probe as non-targeting control^14^.

### RNA FISH

RNA FISH was performed using standard procedures with minor modifications^23^. Briefly, cells were fixed with 4% paraformaldehyde (PFA) for 10 min at room temperature (RT) and permeabilized with 0.1% Triton X-100 in PBS for 10 min at RT. Hybridization buffer (10 ml) contains 1 g dextran sulfate, 10 mg yeast tRNA, 100 ul RNase inhibitor VRC (200 mM stock), 1 ml 20X SSC, 1 ml formamide (deionized), and nuclease-free water to 10 ml final volume. Cells were hybridized with biotin-labeled probes in hybridization buffer at 37 °C for 16 hours, followed by stringent washes with 10% formamide in 2x SSC at 37 °C for 30 min and in 1× SSC at RT for 15 min. Bound probes were then detected by staining with Streptavidin-Cy3 (1:1000). 20X SSC (Thermo Fisher, AM9763), Dextran sulfate sodium salt (sigma, D8906-50G), Yeast tRNA (Invitrogen 15401011), Streptavidin-Cy3 (APEXBIO K1079).

### RNA in situ proximity labeling (FISH+)

The FISH+ method utilizes RNA in situ hybridization to direct proximity biotinylation. The approach commences with the fixation and permeabilization of cells, followed by the hybridization of biotin-labeled probes to the target RNAs. Upon the guidance of these probes, streptavidin-HRP is recruited to the RNA of interest. Crucially, this step is performed prior to any fluorescent staining to avoid competition for biotin sites on the probe. In the presence of biotin-phenol and H_2_O_2_, the tethered streptavidin-HRP catalyzes the in situ biotinylation of proteins proximal to the targeted RNAs. The detailed labelling procedure is as follows: 0.5 mM biotin-phenol (BP) was added to the sample for preincubation. H_2_O_2_ was then added at a final concentration of 1 mM, and the reaction were incubated for 1 min at RT. Then, the labeling medium solution was quickly discarded and the reaction was stopped with cold quenching buffer (10 mM sodium ascorbate, 10 mM sodium azide and 5 mM Trolox). Cells were washed twice with quenching buffer, followed by three washes with PBS. Following the reaction, the workflow diverges based on the downstream analysis: (1) For imaging, cells are stained with streptavidin-Cy3 to visualize RNA-proximal biotinylation, labelling both the biotinylated proteins and RNAs. (2) For proteomics, cells were scraped from 10-cm plate with 1 ml of RIPA buffer. The biotinylated proteins were then affinity-enriched using streptavidin-conjugated beads and analyzed through western blotting or mass spectrometry (MS). Sodium ascorbate (sigma, A4034-1g), trolox (Sigma 238813). We provided a detailed step-by-step procedure in Supplementary protocol.

### Label-free mass spectrometry

Enriched streptavidin beads were washed two times with 1 ml RIPA buffer. Beads were then resuspended and washed once with 1 ml 1 M KCL, 1 ml 0.1 M Na_2_CO_3_, 1 ml 2 M urea in 10 mM Tris-HCl pH 8. Wash once again with 1 ml RIPA. Then enriched streptavidin beads were washed three times with 50 mM Triethylammonium bicarbonate (TEAB, Thermo Scientific). Beads were resuspended in 100 ul 20 mM TEAB dissolved trypsin upon pulldown protein amount, (10-cm culture plate for Hela cells, add trypsin 2.5 μg), 37 °C overnight. Then wash beads with 50 ul 1% formic acid twice, combine together (centrifuge 12,000 g 5 min, aspirate upper to new tube for down step). The digested peptides were desalted with C18 SPE column (WAT054955).

LC-MS/MS was performed on a trapped ion mobility quadrupole time-of-flight mass spectrometer (timsTOF Pro, Bruker) coupled to a nano-HPLC (nanoElute, Bruker Daltonics). 200 ng enriched peptides were loaded in solvent A (0.1% formic acid in water, FA) onto a 250 mm × 75 μm ID C18 analytical column packed with 1.6 μm particles and maintained at 50°C. Peptides were separated at a flow rate of 200 nL/min using a 60 min gradient of solvent B (0.1% FA in acetonitrile): 2–22% B for 45 min, 22–37% B for 5 min, 37–80% B for 5 min, followed by 80% B for 5 min. MS data were acquired in positive ion mode over an m/z range of 300–1700. Ion mobility was scanned over 0.6–1.6 Vs/cm², with TIMS accumulation and ramp times of 50 ms. Data were acquired using parallel accumulation-serial fragmentation (PASEF), with each cycle consisting of one full TIMS-MS scan followed by 10 PASEF MS/MS scans. Collision energy was linearly ramped according to ion mobility, ranging from 20 to 59 eV.

### Mass spectrometry data analysis

The raw mass spectra data was processed using MaxQuant software (version 1.5.5.1) and searched against Uniprot Homo sapiens fata database. The request search resulting ‘proteinGroups.txt’ file was imported into R studio (R-4.0.3-win) and analysed using the DEP package^24^. This package has integrated functionalities including data filtering, variance normalization, imputation of missing values, differential enrichment testing and results visualization. We filtered the data to include proteins identified in all 3 replicates of at least one condition and used default imputation settings (MinProb). Affinity enrichments are performed in triplicate to allow a robust statistical analysis. The list of XIST RNA binding proteins was from reports^5,6,17,19,25^ and starBase v2.0 (https://rnasysu.com/encori/index.php). The list of 45S RNA binding proteins was from literature^6,14,20^, NEAT1 RNA binding proteins was from literature^12,13^. These RNAs are located in cell nucleus, we therefore filtered data using nucleus as “main.location” referred to the human protein atlas project. MS data processing procedure: a. Volcano plot by R: DEP package; b. Filter data: lists of proteins localizing to the nucleolus or nucleus; c. Significantly enrichment: *p*-value < 0.01 and fold change > 2 (Supplementary data 2).

### Western blot

Cell pellets were resuspended in RIPA lysis buffer (50 mM Tris, 150 mM NaCl, 1% Triton X-100, 0.5% sodium deoxycholate, 0.1% SDS, 1× protease inhibitor cocktail (Roche, catalog no. 4693159001), 1 mM PMSF). The suspension was sonicated using a Diagenode Bioruptor® Pico for 10–20 cycles (30 s ON/ 30 s OFF) at 4 °C and 30% amplitude until the lysate was clarified. Following a 10 min incubation on ice, the lysates were centrifuged at 21,000 g for 10 min at 4 °C. The resulting supernatant was collected, and protein concentration was determined using a BCA Protein Assay Kit (catalog no. P0011 Beyotime).

Biotinylated proteins were then affinity-enriched using streptavidin-conjugated beads and analyzed through western blotting^26^. Briefly, 60 μL of Dynabeads MyOne Streptavidin magnetic beads were pre-washed twice with RIPA buffer. The beads were resuspended in 3 ml of RIPA buffer and incubated with clarified lysates for 2 h at 4 °C under gentle rotation. After binding, the beads were collected using a magnetic rack and washed stringently. Biotinylated proteins were then eluted from the beads by adding an equal volume of 4x Laemmli Sample Buffer (Bio-RAD, #1610747) supplemented with 50 mM DTT and 5 mM free biotin, followed by heating at 70 °C for 10 min. The eluted protein samples were separated on a 12% SDS-PAGE gel, and transferred onto nitrocellulose membranes. Membranes were blocked with 1x TBST containing 5% BSA for 30 min and then incubated with Strep-HRP diluted 1: 10,000 for 1 h at RT. After three washes with 1x TBST for 10 min each, signals were detected using enhanced chemiluminescence (ECL) reagent and visualized using a Tanon imaging system (Tanon MINI Space). Antibodies used in this study, Strep-HRP (Beyotime A0303).

### Image data analysis

Hela cell or HEK293T cell nuclei were identified by DAPI staining and used for subsequent analysis (ProLong™ Gold Antifade Mountant with DAPI from Invitrogen, Catalog number: P36931). Streptavidin-Cy3 staining for (APExBIO, Catalog number: K1079). Fluorescence intensity was analysed and quantified using ImageJ software.

### Statistics and reproducibility

Statistical analyses were performed using GraphPad Prism 8. For comparisons between two groups, unpaired two-tailed Student’s t-tests were used in Fig. 2a. For comparisons among multiple groups with a single variable, one-way ANOVA was used in Fig. 2b. Unless otherwise stated, grouped data are presented as mean ± SD from at least *n* = 3 biologically independent experiments. Detailed analyses used for individual experiments are indicated in the corresponding figure legends. *P*-values < 0.05 were considered statistically significant, denoted as *p < 0.05, **p < 0.01, ***p < 0.001, and ****p < 0.0001, “ns” denotes not significant.

### Reporting summary

Further information on research design is available in the Nature Portfolio Reporting Summary linked to this article.

## Supplementary information

Supplementary Information

Description of Additional Supplementary Files

Dateset 1-5

Reporting summary

## Data Availability

The Datasets supporting the conclusions of this study are included within the article and its supplementary files. The FISH oligo probe sets are provided in Suppl. data 1. Quantitative proteomic datasets are provided in Suppl. data 2. Source proteomic data are available via ProteomeXchange with identifier PXD079477. The full heatmap of log2-centered XIST intensity is provided in Suppl. data 3. Source data underlying graphs can be obtained from Suppl. data 4, 5. All other data are available from the corresponding author.
