# Supplementary material for: FISH+ is a ready-to-use proximity labeling method for simultaneous RNA visualization and RNA-interacting protein identification
Source: Commun Biol. 2026 Jun 23;9:1325. doi: 10.1038/s42003-026-10509-0 (PMC13655014; doi:10.1038/s42003-026-10509-0)
Supplement: Supplementary file 1 [file 42003_2026_10509_MOESM1_ESM.pdf]

Supplementary Information

FISH+ is a ready-to-use proximity labeling method for simultaneous RNA visualization and RNA-interacting protein identification

Mingxing Lu[1], Fenglin Shen[2], Yun Liu[3] and Xianghuo He[1]

[1]Fudan University Shanghai Cancer Center and Institutes of Biomedical Sciences, Department of Oncology, Shanghai Medical College, Fudan University, Shanghai 200032, China

[2]Institute of Biomedical Science, Fudan University, Shanghai 200032, China

[3]MOE Key Laboratory of Metabolism and Molecular Medicine, Department of Biochemistry and Molecular Biology, School of Basic Medical Sciences and Shanghai Xuhui Central Hospital, Fudan University, Shanghai 200032, China

[*] **Corresponding Authors:**

Dr. Mingxing Lu, Email: mingxinglu@fudan.edu.cn; or Dr. Xianghuo He, Email: xhhe@fudan.edu.cn, Fudan University Shanghai Cancer Center and Institutes of Biomedical Sciences, Shanghai Medical College, Fudan University, Shanghai 200032, China.

Or Dr. Yun Liu, Email: yliu39@fudan.edu.cn. MOE Key Laboratory of Metabolism and Molecular Medicine, Department of Biochemistry and Molecular Biology, School of Basic Medical Sciences and Shanghai Xuhui Central Hospital, Fudan University, Shanghai 200032, China.

Supplementary materials

Supplementary figure 1. In vitro evaluation of streptavidin-HRP biotinylation labeling activity

Supplementary figure 2. Gene ontology enrichment analysis of 45S and NEAT1 interactomes

Supplementary figure 3. RNase A treatment degrades RNA FISH signals

Supplementary figure 4. FISH+ signals are retained following RNase A treatment

Supplementary figure 5. FISH+ allows RNA visualization by imaging RNA-proximal biotinylated proteins

Supplementary figure 6. FISH+ captures known interactors of PNCTR

Supplementary figure 7. Systematic comparison of FISH+ with existing RNA-centric proteomic studies

Supplementary figure 8. Validation of FISH probes specificity

Supplementary protocol. FISH+ Protocol (RNA-directed proximity labeling)

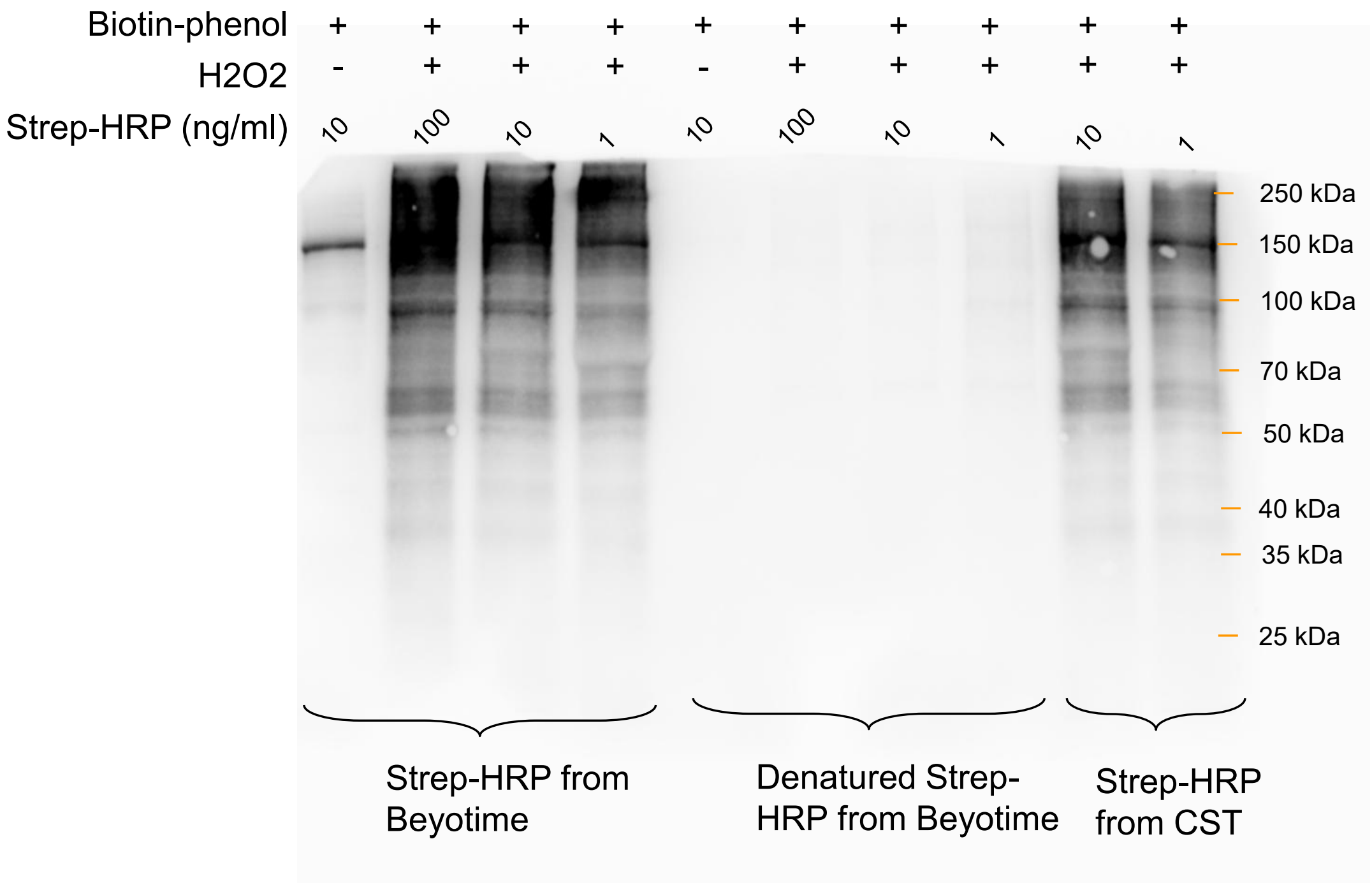

Supplementary figure 1. In vitro evaluation of streptavidin-HRP biotinylation labeling activity

293T cell lysates were diluted to 1 µg/µl and 100 µl aliquots were prepared in 1.5 ml EP tubes. Streptavidin-HRP (Strep-HRP) was added at the indicated concentrations (1, 10, or 100 ng/ml from Beyotime, and 1 or 10 ng/ml from Cell Signaling Technology (CST)). Add biotin-phenol and H_2O_2 to trigger proximity labeling reaction, 1 min later stop the reaction with cold quench buffer. Equal amounts of cell lysate were loaded into each lane. Biotinylation signals were detected with anti-biotin antibody (Beyotime, 1: 1000).

(1) 45S

(2) NEAT1

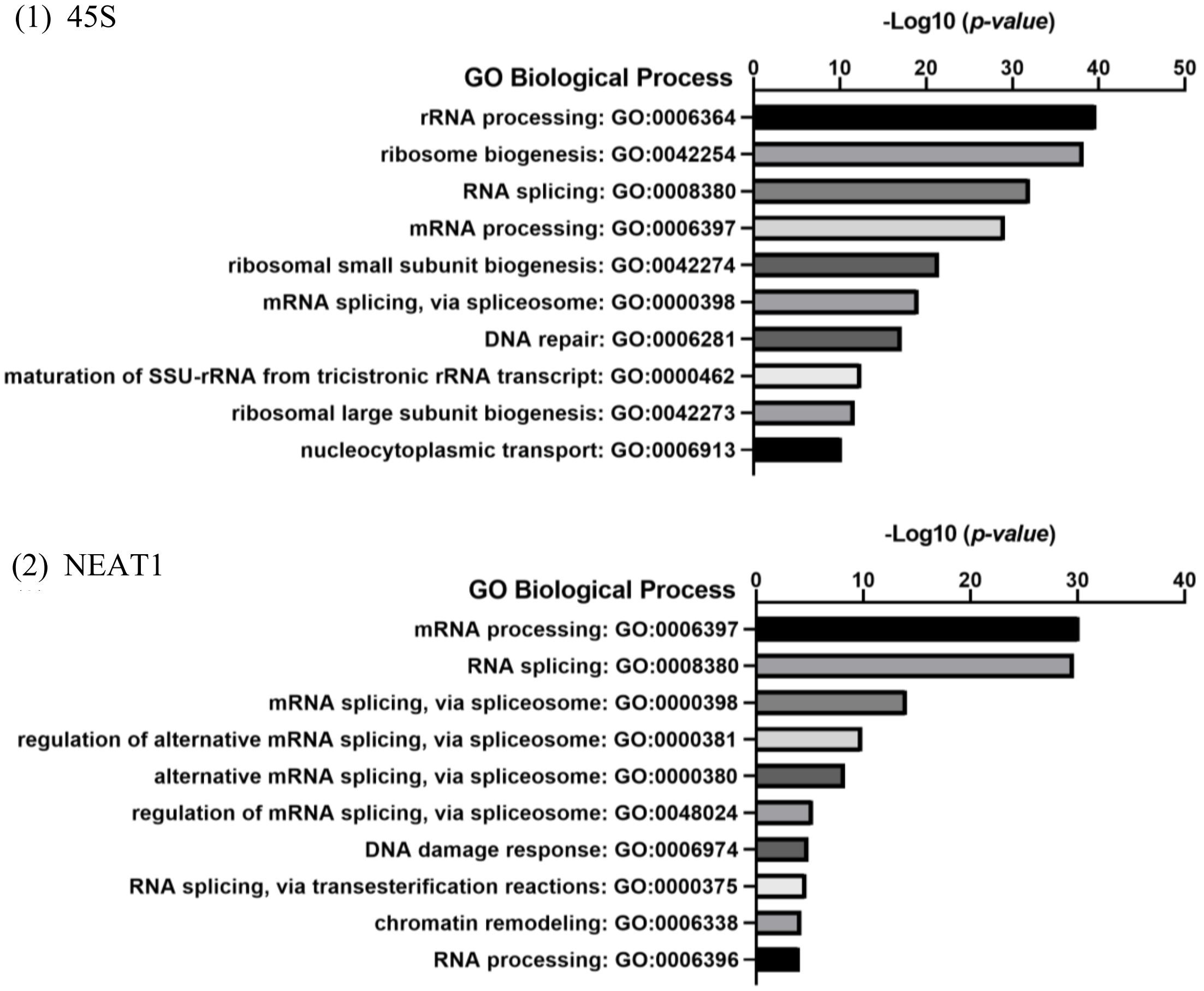

Supplementary figure 2. Gene ontology enrichment analysis of 45S and NEAT1 interactomes

Analysis was performed using DAVID on the subset of enriched proteins. These results reveal a strong enrichment of nucleolus-specific terms for 45S and various RNA processing-related terms for NEAT1. This functional profiling validates the specificity of the FISH+ method.

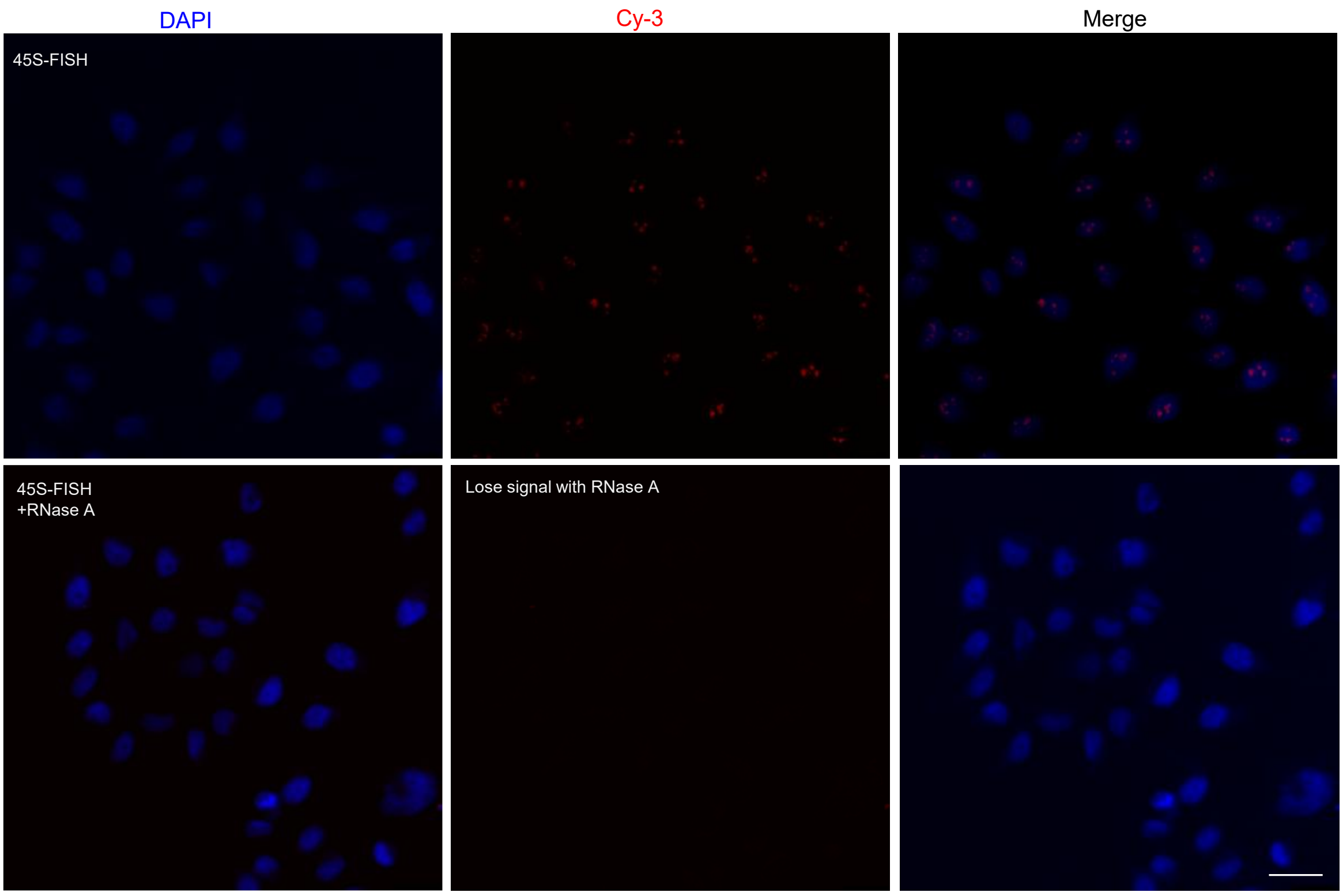

Supplementary figure 3. RNase A treatment degrades RNA FISH signals.

Treatment with RNase A degrades target RNAs, resulting in the loss of FISH signals. Top: conventional RNA FISH targeting 45S RNA (red dots). Bottom: 45S RNA FISH following RNase A treatment, showing the complete disappearance of FISH signals. Nuclei were stained with DAPI (blue). Scale bars: 50 µm.

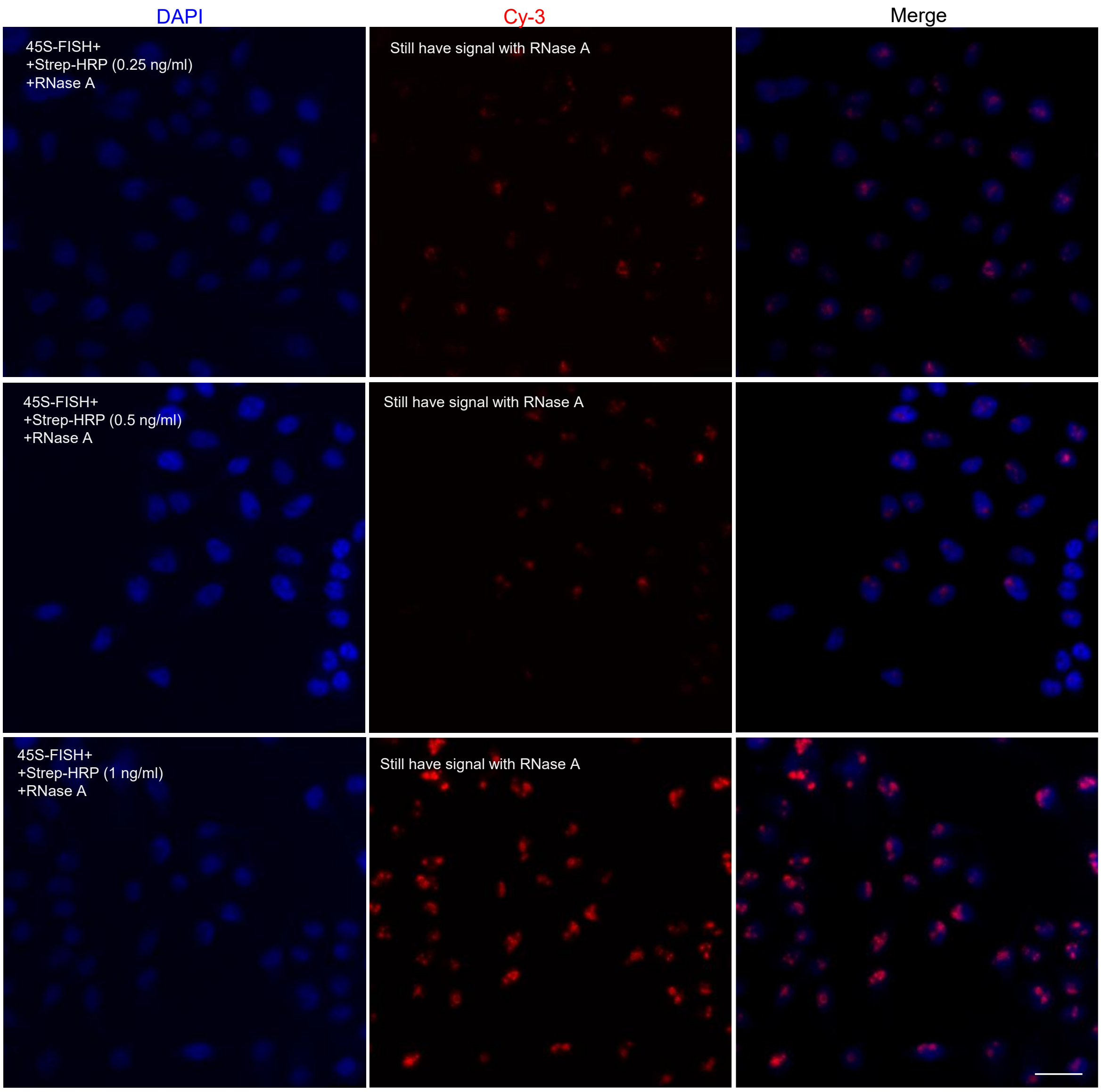

Supplementary figure 4. FISH+ signals are retained following RNase A treatment.

The labeling signal intensity increases in a dose-dependent manner with higher concentrations of the proximity labeling enzyme (Strep-HRP from 0.25 to 1 ng/ml). Notably, while RNase A treatment degrades RNAs, the biotinylated protein signals are preserved. This preservation suggests that the FISH+ method induces proximity biotinylation of both the target RNAs and their closely associated proteins. Signal intensity correlated with the dosage of proximity labeling enzyme. Scale bars: 50 µm.

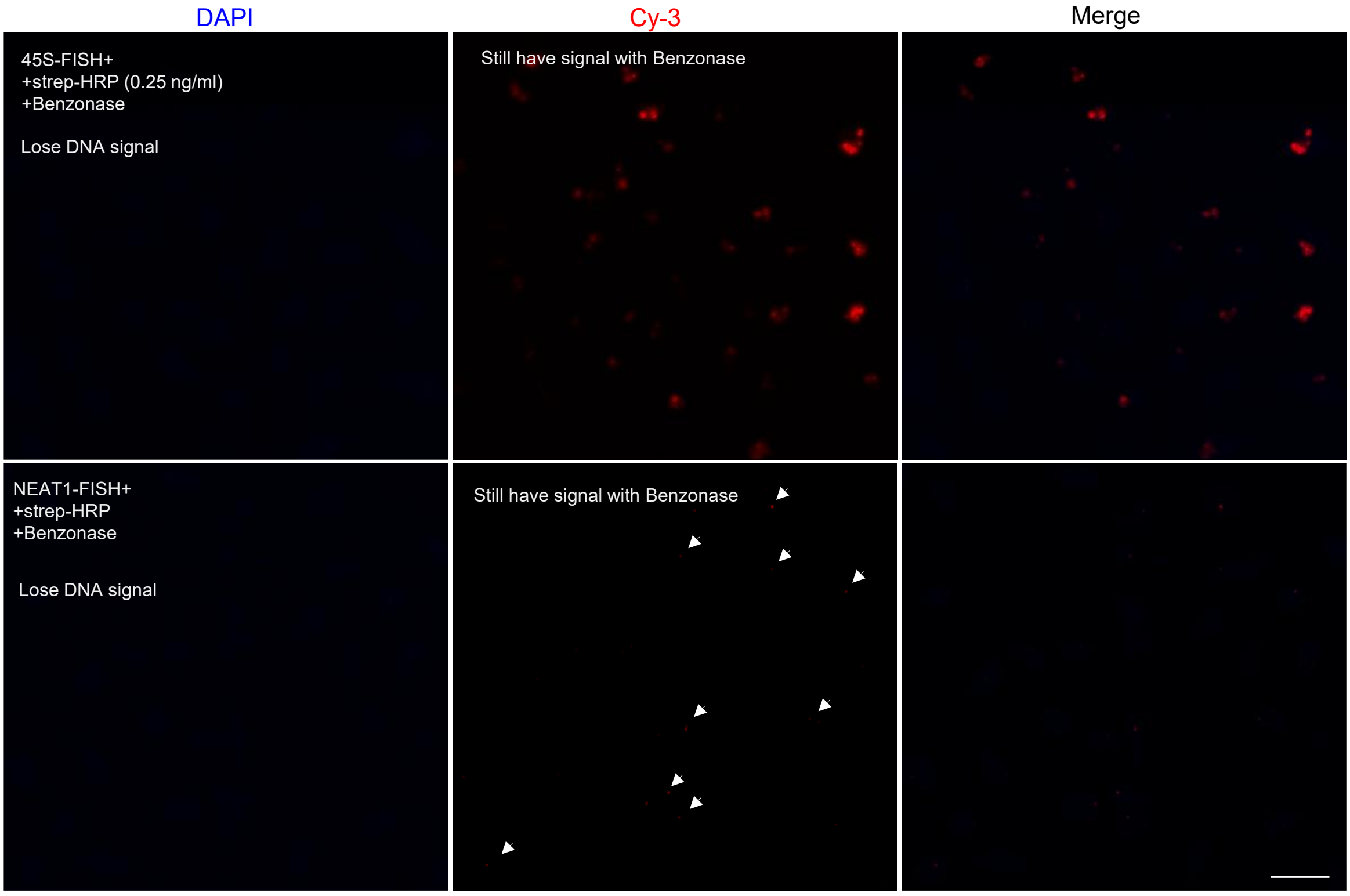

Supplementary figure 5. FISH+ allows RNA visualization by imaging RNA-proximal biotinylated proteins.

Benzonase is an endonuclease that can degrade all types of DNA and RNA. We utilized Benzonase to remove nucleic acids as DAPI staining signals for 45S and NEAT1 disappeared. However, dot signals were still detectable via streptavidin-Cy3 in fixed Hela cells. For both 45S and NEAT1 FISH+, these residual signals confirm that dot patterns observed originate from RNA-proximal biotinylated proteins, even after nucleic acid degradation. This indicates that the FISH+ method successfully labels RNA-proximal proteins, providing insight into RNA-protein interactions. Scale bars: 50 µm.

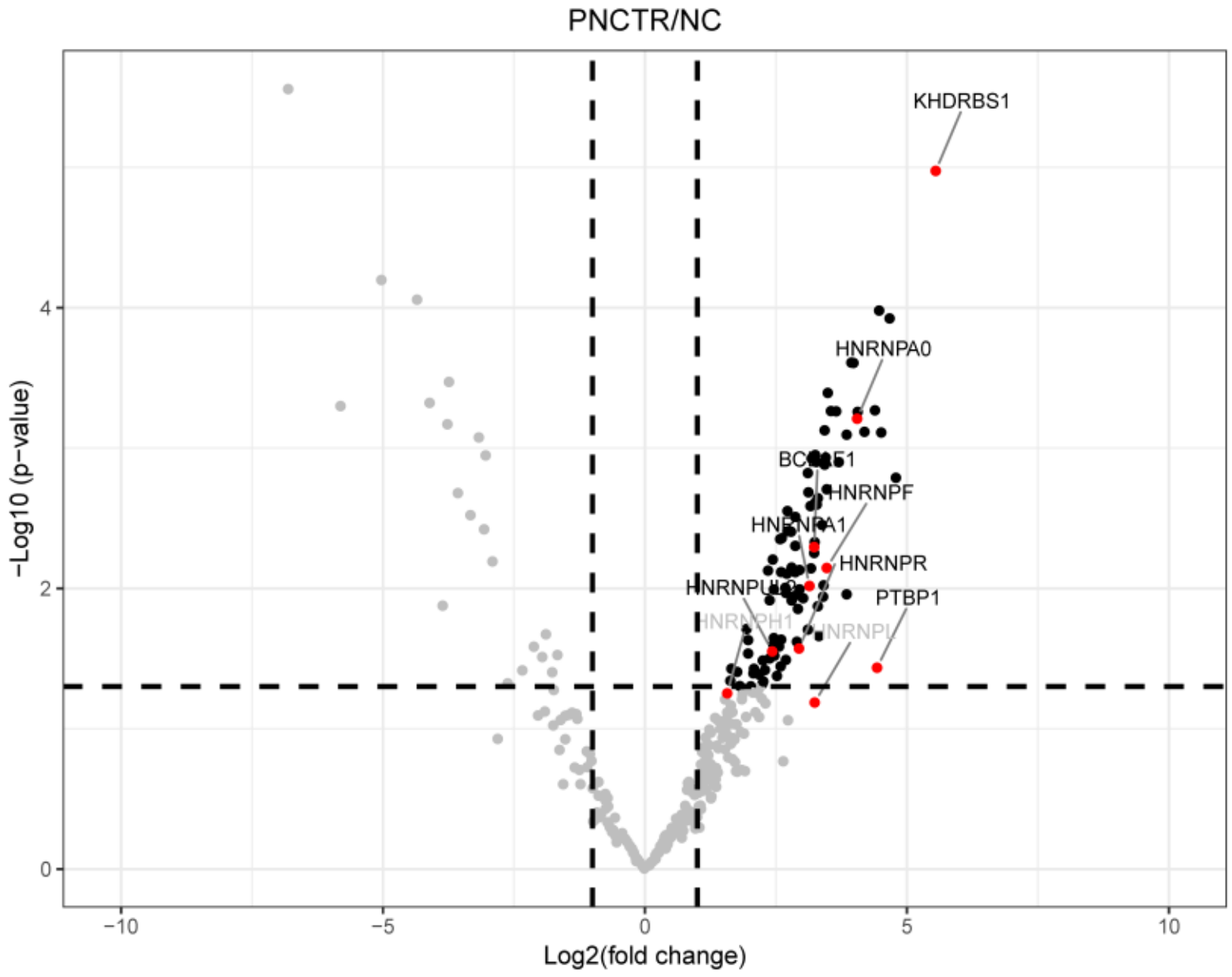

Supplementary figure 6. FISH+ captures known interactors of PNCTR.

Volcano plot showing proteins significantly enriched in PNCTR-targeted samples versus scrambles. The established PNCTR binding partner PTBP1 is clearly captured. Furthermore, FISH+ identified other known interactors, including HNRNPL and KHDRBS1, confirming the sensitivity of the method for transcripts with medium-abundance RNA (~36 copies/cell). Enriched proteins: log2FC>1, p-value<0.05.

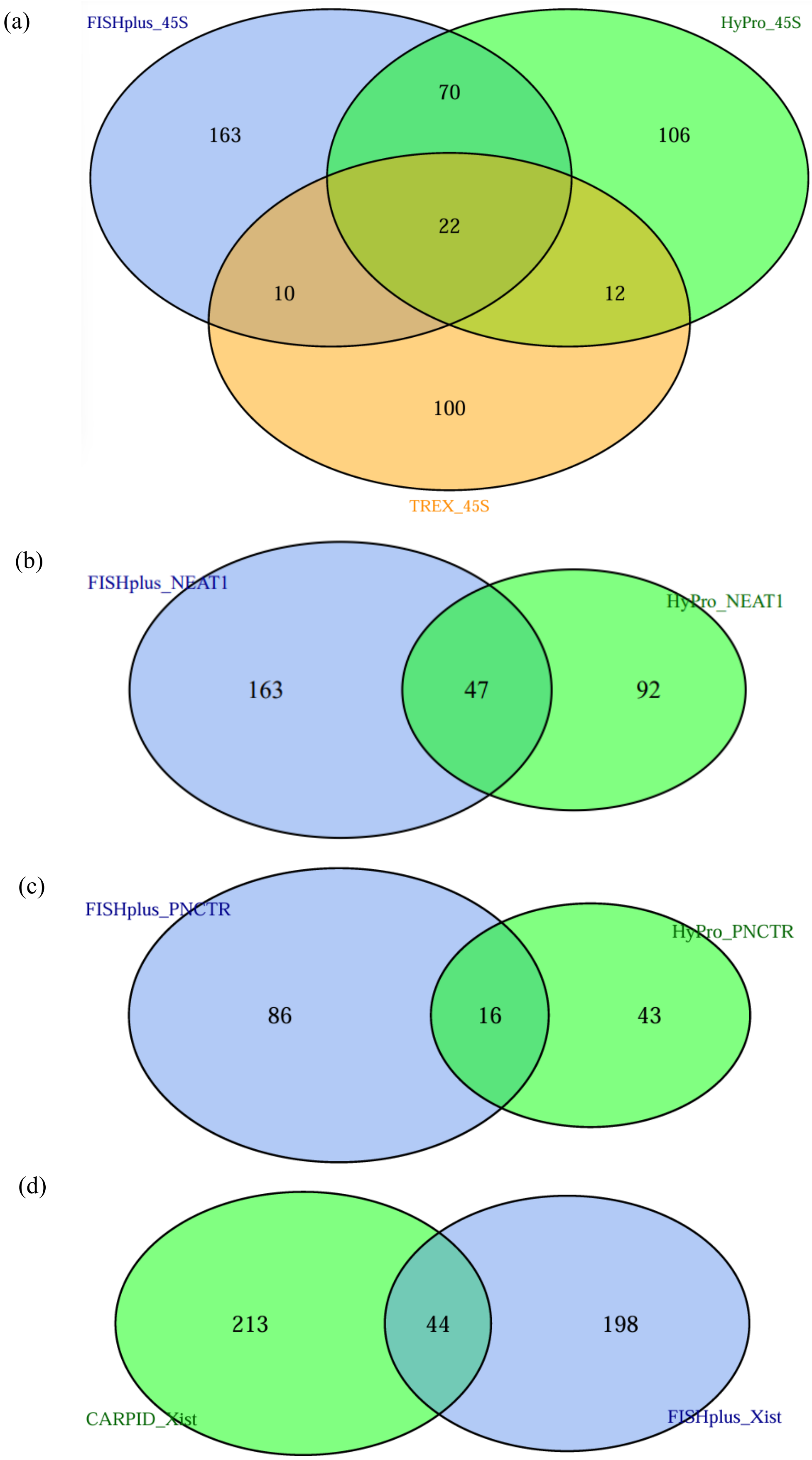
(a)
FISHplus_45S
HyPro_45S
163
70
106
22
10
12
100
TREX_45S
(b)
FISHplus_NEAT1
HyPro_NEAT1
163
47
92
(c)
FISHplus_PNCTR
HyPro_PNCTR
86
16
43
(d)
213
44
198
CARPID_Xist
FISHplus_Xist

Supplemental Figure 7. Systematic comparison of FISH+ with existing RNA-centric proteomic studies.

Venn diagrams illustrate the overlap of significantly enriched proteins identified by FISH+ (FISHplus) compared to published datasets (HyPro, TREX, and CARPID) targeting 45S rRNA, NEAT1, PNCTR, and XIST. (a) Comparison of 45S-interacting proteins. FISH+ identified 265 proteins, overlapping with 92 from HyPro (210 total) and 32 from TREX (114 total). 22 proteins were shared across all three methods. (b-c) Overlap analysis for well-studied lncRNAs showing 47 shared proteins for NEAT1 with HyPro method, 16 for PNCTR. (d) Similarly, there are 44 proteins shared between FISH+ and CARPID method. Significance of enrichment: p-value<0.05, fold change>2.

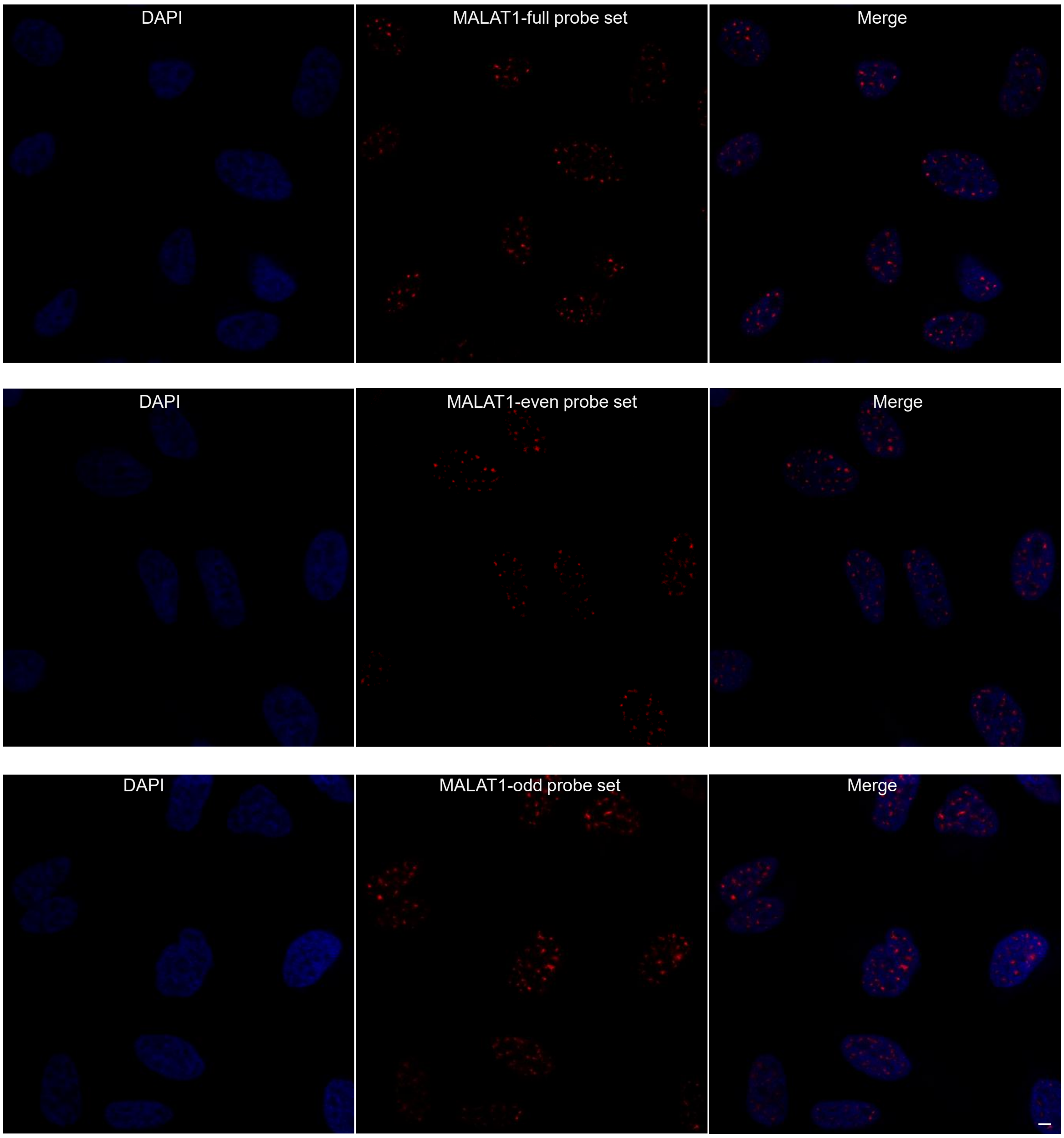

Supplementary figure 8. Expression pattern of even and odd probe subsets of MALAT1

Clustered puncta signals in nuclei from two subsets are roughly equal with the full probe set. Such similar signal pattern suggests that probe design of MALAT1 is specific. Concentration of probe set: 100 nM. Scale bars: 10 µm.

FISH+ Protocol (RNA-Directed Proximity Labeling)

Part I: Hybridization & proximity Labeling

1. Fixation & Permeabilization

- **Fix:** Incubate cells with 4% paraformaldehyde (PFA) for 10 min at room temperature (RT). Permeabilize cells with 0.1% Triton X-100 in PBS for 10 min at RT.
  Alternative method for fixation and permeabilization: Fix cells with 0.5 mg/mL Dithiobis(succinimidyl propionate) (DSP), followed by permeabilization in 70% ethanol. (Yap et al Mol Cell 2022)
- **Wash:** twice with PBS, 5 min each.

2. Probe Hybridization

- **Pre-Hybridization:** incubate in 10% formamide / 2x SSC for 10 min at RT.
- **Hybridization:** Dilute probes and denature at 75°C for 5 min; Incubate cells with biotinylated probes (5 nM for 45S, 25 nM for NEAT1, 50 nM for XIST) in Hybridization Buffer (2xSSC, 10% formamide, 10% dextran sulfate). Hybridize at 37°C for 16 hours.

3. Wash and Blocking

- **Wash:** Stringent washes (10% formamide/2× SSC at 37°C, 30 min; 1× SSC at RT 15 min).
- **Blocking:** Block with 4× SSC supplemented with RNasin at RT, 30 min.

4. HRP Recruitment

- **Incubation:** Incubate with Streptavidin-HRP (10 ng/mL) in 4x SSC + RNasin for 1 hour at RT.
- **Wash:** wash sequentially with 4x SSC for 10 min, 4x SSC/0.1% Triton X-100 for 10 min, and 4x SSC for 10 min.

5. Proximity Biotinylation

- **Equilibration:** Cells are incubated in PBS for 10 min.
- **Labeling:** 0.5 mM Biotin-Phenol (BP), 1 mM H_2O_2 is added to initiate the reaction for exactly 1 minute at RT.
- **Quenching:** The reaction is stopped immediately by exchanging the solution with Quencher Buffer (DPBS containing 10 mM sodium azide, 10 mM sodium ascorbate, 5 mM Trolox). Cells are washed 3 times with cold Quencher Buffer.

Part II: Downstream Application (Choose Path)

Option A: Fluorescence Imaging

- ***Stain:*** *Incubate cells with Streptavidin-Cy3 to visualize RNA-proximal biotinylation.*
- ***Image:*** *Perform fluorescence microscopy (visualizes both biotinylated proteins and RNA location).*

Option B: Proteomics (Mass Spectrometry)

- ***Proceed to Step 6 below.***

Part III: Proteomic Sample Preparation (Path B Only)

6. Cell Lysis

- Cells are scraped in10-cm dish with 1 mL RIPA Lysis Buffer (supplemented with quenchers). The lysate is sonicated in 300 ul aliquots for 10 cycles with 1.5-mL microfuge tubes.
- After sonication, boiled at 95°C for 1 hour to reverse crosslinks.
- Centrifuge at 16,000 × g for 10 min at 4°C. Collect the supernatant.

7. Enrichment

- Incubate the supernatant lysate with Streptavidin Magnetic Beads for 2 hours at 4°C.

8. Stringent Washing

- Wash beads sequentially (1 mL each):

    1. 1M KCl.
    2. 0.1 M Na_2CO_3
    3. 2 M Urea in 10 mM Tris-HCl pH 8.0
    4. RIPA buffer

9. On-Bead Digestion (MS)

- **Digest:** Wash beads with Triethylammonium bicarbonate (TEAB). Add Trypsin (1–2.5 μg) and incubate overnight at 37°C.
- **Elute:** Elute peptides with 1% Formic Acid.
- **Finish:** Dry (SpeedVac) and Desalt (C18 StageTip) for LC-MS/MS.
