# Supplementary material for: FISH+ is a ready-to-use proximity labeling method for simultaneous RNA visualization and RNA-interacting protein identification
Source: Commun Biol. 2026 Jun 23;9:1325. doi: 10.1038/s42003-026-10509-0 (PMC13655014; doi:10.1038/s42003-026-10509-0)
Supplement: Supplementary file 2 [file 42003_2026_10509_MOESM2_ESM.pdf]

**Description of Additional Supplementary File**

Supplementary data 1. FISH oligo probe sets

Supplementary data 2. Proteomic datasets

Supplementary data 3. Log2-centered intensity heatmap of XIST

Supplementary data 4. The quantified signal intensity of FISH and FISH+

Supplementary data 5. The quantified signal intensity of RNA labeling increases with higher enzyme concentrations.
