# Supplementary material for: FISH+ is a ready-to-use proximity labeling method for simultaneous RNA visualization and RNA-interacting protein identification
Source: Commun Biol. 2026 Jun 23;9:1325. doi: 10.1038/s42003-026-10509-0 (PMC13655014; doi:10.1038/s42003-026-10509-0)
Supplement: Supplementary file 4 [file 42003_2026_10509_MOESM3_ESM.zip › Supplementary Data/Supplementary Data 3.pdf]

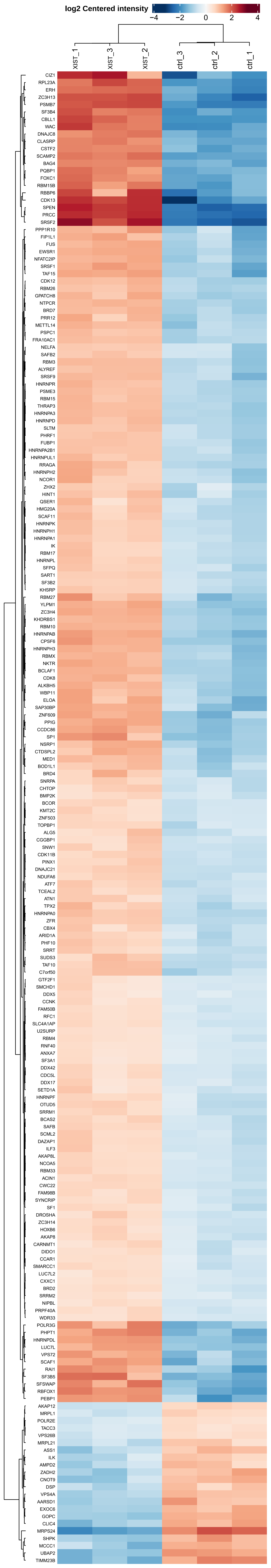
log2 Centered intensity
−4
−2
0
2
4
XIST_1
XIST_3
XIST_2
ctrl_3
ctrl_2
ctrl_1
CIZ1
RPL23A
ERH
ZC3H13
PSMB7
SF3B4
CBLL1
WAC
DNAJC8
CLASRP
CSTF2
SCAMP2
BAG4
PQBP1
FOXC1
RBM15B
RBBP6
CDK13
SPEN
PRCC
SRSF2
PPP1R10
FIP1L1
FUS
EWSR1
NFATC2IP
SRSF1
TAF15
CDK12
RBM26
GPATCH8
NTPCR
BRD7
PRR12
METTL14
PSPC1
FRA10AC1
NELFA
SAFB2
RBM3
ALYREF
SRSF9
HNRNPR
PSME3
RBM15
THRAP3
HNRNPA3
HNRNPD
SLTM
PHRF1
FUBP1
HNRNPA2B1
HNRNPUL1
RRAGA
HNRNPH2
NCOR1
ZHX2
HINT1
QSER1
HMG20A
SCAF11
HNRNPK
HNRNPH1
HNRNPA1
IK
RBM17
HNRNPL
SFPQ
SART1
SF3B2
KHSRP
RBM27
YLPM1
ZC3H4
KHDRBS1
RBM10
HNRNPAB
CPSF6
HNRNPH3
RBMX
NKTR
BCLAF1
CDK8
ALKBH5
WBP11
ELOA
SAP30BP
ZNF609
PPIG
CCDC86
SP1
NSRP1
CTDSPL2
MED1
BOD1L1
BRD4
SNRPA
CHTOP
BMP2K
BCOR
KMT2C
ZNF503
TOPBP1
ALG5
CGGBP1
SNW1
CDK11B
PINX1
DNAJC21
NDUFA6
ATF7
TCEAL2
ATN1
TPX2
HNRNPA0
ZFR
CBX4
ARID1A
PHF10
SRRT
SUDS3
TAF10
C7orf50
GTF2F1
SMCHD1
DDX5
CCNK
FAM50B
RFC1
SLC4A1AP
U2SURP
RBM4
RNF40
ANXA7
SF3A1
DDX42
CDC5L
DDX17
SETD1A
HNRNPF
OTUD5
SRRM1
BCAS2
SAFB
SCML2
DAZAP1
ILF3
AKAP8L
NCOA5
RBM33
ACIN1
CWC22
FAM98B
SYNCRIP
SF1
DROSHA
ZC3H14
HOXB6
AKAP8
CARNMT1
DIDO1
CCAR1
SMARCC1
LUC7L2
CXXC1
BRD2
SRRM2
NIPBL
PRPF40A
WDR33
POLR3G
PHPT1
HNRNPDL
LUC7L
VPS72
SCAF1
RAI1
SF3B5
SFSWAP
RBFOX1
PEBP1
AKAP12
MRPL1
POLR2E
TACC3
VPS26B
MRPL21
ASS1
ILK
AMPD2
ZADH2
CNOT9
DSP
VPS4A
AARSD1
EXOC6
GOPC
CLIC4
MRPS24
SHPK
MCCC1
UBAP2
TIMM23B
